# A Method for Medical Diagnosis Based on Optical Fluence Rate Distribution at Tissue Surface

**DOI:** 10.3390/ma10091104

**Published:** 2017-09-20

**Authors:** Omnia Hamdy, Jala El-Azab, Tarek A. Al-Saeed, Mahmoud F. Hassan, Nahed H. Solouma

**Affiliations:** 1 Department of Engineering Applications of Laser, National Institute of Laser Enhanced Sciences (NILES), Cairo University, Giza Governorate 12613, Egypt; jala@niles.edu.eg (J.E.-A.); nsolouma@niles.edu.eg (N.H.S.); 2 Department of Biomedical Engineering, Faculty of Engineering, Helwan University, Cairo Governorate 11795, Egypt; Tarek1971@ieee.org; 3 Department of Basic Sciences, Faculty of Engineering, Benha University, Qalubiya Governorate 13511, Egypt; mahmoudfathy.electron@yahoo.com; 4 Department of Biomedical Engineering, Faculty of Engineering, King Faisal University, Al-Ahsa 31982, Saudi Arabia

**Keywords:** optical fluence rate, tissue optical parameters, Kubelika-Munk model, diffuse reflection, finite element method, diffusion equation

## Abstract

Optical differentiation is a promising tool in biomedical diagnosis mainly because of its safety. The optical parameters’ values of biological tissues differ according to the histopathology of the tissue and hence could be used for differentiation. The optical fluence rate distribution on tissue boundaries depends on the optical parameters. So, providing image displays of such distributions can provide a visual means of biomedical diagnosis. In this work, an experimental setup was implemented to measure the spatially-resolved steady state diffuse reflectance and transmittance of native and coagulated chicken liver and native and boiled breast chicken skin at 635 and 808 nm wavelengths laser irradiation. With the measured values, the optical parameters of the samples were calculated in vitro using a combination of modified Kubelka-Munk model and Bouguer-Beer-Lambert law. The estimated optical parameters values were substituted in the diffusion equation to simulate the fluence rate at the tissue surface using the finite element method. Results were verified with Monte-Carlo simulation. The results obtained showed that the diffuse reflectance curves and fluence rate distribution images can provide discrimination tools between different tissue types and hence can be used for biomedical diagnosis.

## 1. Introduction

Light propagation in any turbid media as biological tissue is affected by its optical properties. The main optical parameters of tissues are absorption coefficient $\mu_{a}$, scattering coefficient $\mu_{s}$ and anisotropy g. These parameters are highly wavelength-dependent and related to many physiological changes in tissues. Absorption measurements are directly related to tissue hemoglobin and melanin concentration as well as tissue oxygen saturation, while, scattering measurements are connected with lipid concentration, cell nucleus size and cell membrane refractive index change. Therefore, scattering and absorption properties are very important indicators for tissue health and help in diagnosis procedures [1].

When dealing with biological tissue, near infrared light (600 to 900 nm) is preferred because of its high penetration depth and high penetration means more information about the examined tissue [1]. Upon illuminating tissue with NIR light, the detected transmitted and or reflected signal become diffusive. Diffuse reflectance and transmittance values are the key data in tissue optical parameters estimation process [2].

Instead of common Maxwell’s equations, radiative transport equation RTE is used to describe light propagation in biological tissues due to the denes and multiple scattering nature of turbid media such as biological tissues. These tissues’ special characteristics creates very complex waves superposition and boundary conditions that make it so hard to use Maxwell’s equations in describing light propagation in tissues [1,2]. However, RTE is considered an approximation to Maxwell’s equations based of conversation of energy, describing photons propagation in tissues through space and time [2]. Some assumptions like diffusion approximation have been made to obtain an analytical and/or numerical solution of RTE [2].

Many experimental and numerical methods can be employed to obtain tissue optical parameters from measured values of total diffuse reflectance, total diffuse transmittance and collimated transmittance measurements based on solutions of the RTE. These methods include inverse Monte-Carlo simulation, inverse adding-doubling method and Kubelka-Munk model [2]. Kubelka-Munk model remains the least complicated model and is widely used for medical purposes [3,4].

Measurements of reflectance and transmittance could be implemented experimentally by two common techniques. In the first technique, a single (or double) integrating sphere is used to integrate and redirect the light to be measured [5,6]. While in the second method, a distant detector scans the tissue to obtain a spatially-resolved diffuse reflectance and transmittance data [7]. The spatially-resolved steady-state diffuse reflectance curve is considered a fingerprint of an examined tissue [6], therefore, it can be employed to differentiate normal from abnormal tissue, and hence, used for diagnosis purpose.

Because of its main relation with tissue physiological changes, estimation of tissues optical parameters became a very interesting area for biomedical engineers and scientists. In 1993, Prahl et al. presented a method for estimating tissue optical parameters during heating with an argon laser [8]. In their experiment, diffuse reflection and transmission have been measured using double integrating spheres. The sample was located between the spheres and the photodiode was located about one meter from the sample to ensure that most of the light collected was collimated transmitted light. The accuracy of this method was 10% for all optical properties of tissue in the case of sample thicknesses exceeding one optical depth.

The optical parameters of bovine muscle, adipose, and liver tissue, as well as chicken muscle were obtained from remote measurements of spatially resolved absolute diffuse reflectance [9]. The method was based on a neural network trained on the results of Monte-Carlo simulations. The experimental setup was based on a laser source and a CCD camera. Another method for measuring the diffuse reflection of a sample was presented by Bolt et al. who used a two-dimensional CCD camera with 14-bit dynamic range and a spatial resolution down to 20 μm [10]. The volume-reflection of the sample was measured without the need to scan the sample surface. This setup was presented to be suitable for measuring close to the illuminating beam and its results were compared to the previous results of Monte-Carlo and diffusion theories.

A prototype frequency domain optical imaging device for breast cancer detection was designed by Brian et al. [11]. The device employed radio-frequency intensity modulated near infrared light to quantitatively image both the scattering and absorption coefficients of tissue. The devices used a laser diode and a photomultiplier tube, which are multiplexed automatically through 32 large core fiber optic bundles using high precision linear translation stages. The performance of the system is dependent upon the characteristics of the modulated light source and detector.

An endoscopic measurement of optical properties of Intralipid-dye tissue phantoms with temperature-controlled 675 nm laser diode was also performed in [12]. Diffuse reflectance measurements were obtained with fiber optic probe and Monte-Carlo method was used to calculate the radial distributions of a given optical properties pair.

Another method was used by Dimofte et al. [13] to predict the tissue optical parameters by measuring the ratio of light fluence rate to source power along a linear channel at a fixed distance from an isotropic point source. Isotropic detector was used to collect diffused reflected light and the detector position is determined by a computer-controlled step motor. The results have been then fitted with a diffusion equation to determine absorption coefficient and scattering coefficient.

In 2006, Chandra et al. [14] developed a method to quantitatively characterize thick biological tissues non-invasively by combining both experimental and computational approaches in tissue optical spectroscopy. They developed and employed a prototype instrumentation for reflectance and fluorescence spectroscopy that may be used clinically and also in experimental studies. They also developed novel computational codes to model time-resolved excitation and fluorescent light propagation in multifluorophore biological tissues. The system presented by Chandra was later improved by Wilson et al. in [15] to make a photon-tissue interaction (PTI) model to analyze a number of 96 pairs of reflectance and fluorescence spectra from freshly excised human pancreatic tissues. For each pair of spectra, the PTI model extracted a cellular nuclear size parameter from the measured reflectance and the relative contributions of extracellular and intracellular fluorophores to the intrinsic fluorescence.

Three fiber-based diffuse reflection spectroscopy has been employed to characterize the absorption and scattering properties of tissue-mimicking liquid phantoms using tungsten halogen lamp and an in-house LED module between 400 and 700 nm [16]. The detector used is a charge-coupled device (CCD) array-based spectrometer. The optical parameters were extracted with errors less than 10%.

The diffuse imaging method was then improved by O’Sullivan et al. [17] as they made a combination between the principle of frequency domain photon migration and near-infrared spectroscopy to improve the spectral bandwidth. This system is called diffuse optical spectroscopic imaging (DOSI). Using this system, they obtained the absorption and scattering properties at spectra (650–1000 nm). The main problem of DOSI device is its high cost (~$50,000 to $75,000) due to the presence of the FDPM (frequency domain photon migration) functionality. The DOSI device consists of a network analyzer, laser driver and bias network.

Diffuse reflectance imaging has been also used in studying the absorber heterogeneity effect on light propagation in tissues [18]. A solid scattering phantom with an absorber inclusion was used as the sample; and the light source was a 660 nm laser diode. A CMOS camera was used to capture an image of the light distribution at the top surface of the sample.

In this work, we propose one more optical method to differentiate biological tissue based on fluence rate distribution. The optical fluence rate distribution depends on the optical tissue parameters and hence displaying and investigating such distribution can provide a diagnostic tool. This work presents another medical diagnostic tool that is based of optical fluence rate distribution images rather than previous methods that depend on optical parameters values, this provide an image-based diagnostic tool which is usually preferred by physicians. This method could be helpful in monitoring tissue physiological changes through some medical processes like bio-stimulation or photodynamic therapy.

To obtain the fluence rate distribution, we used the distant-detector method in an ex vivo study to measure the steady sate diffuse reflectance and diffuse transmittance as well as collimated transmittance for different tissue samples. The measured values were provided to the Kubelka-Munk model to calculate the values of the absorption coefficient µa, the scattering coefficient µs and the anisotropy g. Laser types used in this work are He-Ne laser at 635 nm and semiconductor laser diode at 808 nm.

In an intermediate step, the obtained values of optical parameters were entered into Monte-Carlo modeling of light transport in multilayered tissue (MCML) code to predict the corresponding diffuse reflectance and transmittance [4]. This step is used as a performance evaluation and validation. The obtained optical parameters were then used in the diffusion equation to predict the fluence rate distribution at the surface of the samples using the finite element method.

## 2. Materials and Methods

In this work, diffuse reflectance and transmittance of biological samples were measured experimentally using a distant detector-based setup and the optical parameters were estimated using Kubelka-Munk model then the diffusion equation was used to obtain the fluence rate distribution at the sample boundary. The fluence rate distributions were then investigated and analyzed to extract discriminant features that can be used to differentiate tissue types.

### 2.1. Sample Preparation

In this study two types of tissues: namely chicken liver and chicken skin, were used in normal and coagulated conditions. The normal chicken liver used in the normal condition has a slice of 2 mm thickness. The sample was frozen for 24 h after the chicken was slaughtered. To make a coagulated chicken liver sample, the sample was boiled for 3 min in water at 95 °C. Samples from chicken skin of 1 mm thickness were also prepared in normal and boiled conditions to make the measurements.

### 2.2. Experimental Setup

**Figure 1:**
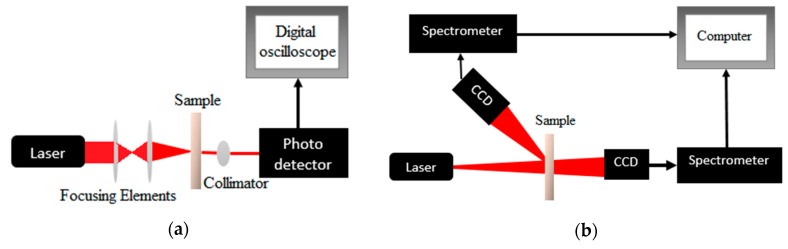
The schematic diagram of the proposed setup: (**a**) collimated transmission measurement; (**b**) diffuse reflection and transmission measurement.

The measurements of the diffuse reflection, diffuse transmission and collimated transmittance of the samples have been taken using a distant detector-based method. When measuring the collimated transmission, a focusing set of lenses has been used to focus the input laser beam. A lens has been used to collimate the light transmitted from the sample. The collimated light is then received by a photodetector which sends its output to a digital oscilloscope as shown in Figure 1a. A CCD (TCD1304AP) has been fixed on a transition stage with a micrometer in order to make spatially-resolved measurements of diffuse reflection and transmission. The CCD is connected to STDFSM digital fiber spectrometer that is connected to a computer for storage and processing of the results as shown in Figure 1b. The experiments has been ran with 1 mm spatial step and continue to scan up to 10 mm.

### 2.3. Mathmatical Analysis

Kubelka-Munk method has been used to estimate the optical parameters which have been fed to the diffuse equation to obtain the fluence rate distribution.

#### 2.3.1. Kubelka-Munk Model

**Figure 2:**
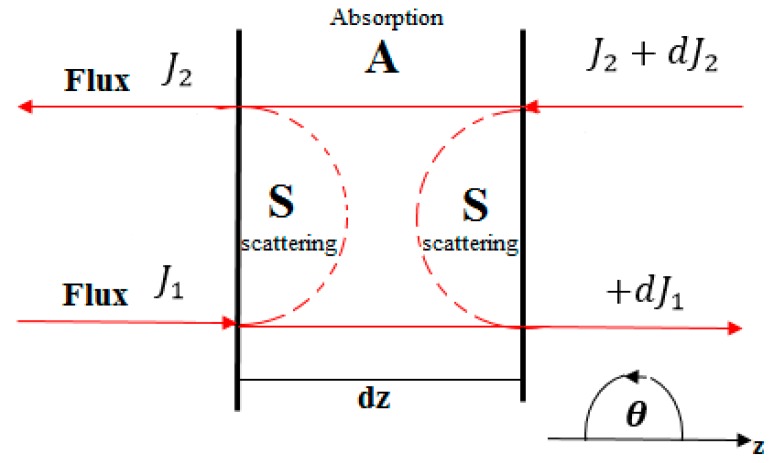
The Geometry of two fluxes in Kubelka-Munk theory.

The Kubelka-Munk method has been widely used to separate light attenuation due to absorption from that due to scattering in turbid media such as biological tissues. The model is employed to determine absorption coefficient µa and reduced scattering coefficient ${\overset{‵}{\mu }}_{s}$ from the measured values of total diffuse reflectance Rd, diffuse transmittance Td and collimated transmittance Tc. This method is based on two propagating fluxes inside the tissue as illustrated in Figure 2: one flux in the direction of the incident beam $J_{1}$ and the other in the backscattered direction $J_{2}$ [2,19].

Two Kubelka-Munk coefficients, S and A, are defined for the absorption and scattering of diffuse radiation, respectively. With these parameters, two differential equations can be formed:

(1) $$\frac{dJ_{1}}{dz}=-S\;J_{1}-A\;J_{1}+S\;J_{2}$$

(2) $$\frac{dJ_{2}}{dz}=-S\;J_{2}-A\;J_{2}+S\;J_{1}$$

where z refers to the direction of the incident radiation and the general solution of Equations (1) and (2) can be expressed by:

(3) $$J_{1}\left(z\right)=c_{11}\;e^{-\gamma z}+c_{12}\;e^{+\gamma z}$$

(4) $$J_{2}\left(z\right)=c_{21}\;e^{-\gamma z}+c_{22}\;e^{+\gamma z}$$

According to Kottler [20], the Kubelka-Munk coefficients are related to diffuse reflectance and transmittance as follows:

(5) $$R_{d}=\frac{\sinh(Sb\;D)}{a\;\cosh\left(S\;b\;D\right)+b\;\sinh(S\;b\;D)}$$

(6) $$T_{d}=\frac{b}{a\;\cosh\left(S\;b\;D\right)+b\;\sinh(S\;b\;D)}$$

where D is the optical depth of the examined slap in the tissue sample. The parameters *a* and *b* can be expressed in terms of:

(7) $$a=\frac{1+R_{d}^{2}-T_{d}^{2}}{2R_{d}}$$

(8) $$b=\sqrt{a^{2}-1}$$

The fraction of loss in flux due to absorption by unit path length is called *A* and fraction due to scattering is denoted as *S*, where *A* and *S* are related to *R_d_* and *T_d_* as [2,19]:

(9) $$S=\frac{1}{bd}\ln\left[\frac{1-R_{d}\left(a-b\right)}{T_{d}}\right]$$

(10) A=(a−1)S

then the relation of *S* and *A* to the scattering and absorption coefficients can be expressed as [2,19]:

(11) $$A=2\mu_{a}$$

(12) $$S=\frac{3}{4}\mu_{s}\left(1-g\right)-\frac{1}{4}\mu_{a}$$

where $\mu_{s}\left(1-g\right)={\overset{‵}{\mu }}_{s}$ is called the reduced scattering coefficient

Total attenuation coefficient $\mu_{t}=\mu_{a}+\mu_{s}$ of the sample can be obtained from collimated transmittance measurements using Bouguer-Beer-Lambert law as [7]:

(13) $$T_{c}=e^{-\mu_{t}d}$$

where d is the thickness of the examined sample.

From Kubelka-Munk calculations, absorption and reduced scattering coefficient can be obtained, then from (14) the total attenuation coefficient can be determined and after that the scattering coefficient can be calculated from $\mu_{s}=\mu_{t}+\mu_{a}$. Upon knowing μ and ${\overset{‵}{\mu }}_{s}$, the anisotropy g could be obtained. Thus, the main three optical parameters ($\mu_{a}$, $\mu_{s}$, g) of the sample can be calculated from the experimental measurements of $R_{d}$, $T_{d}$ and $T_{c}$.

#### 2.3.2. Diffusion Equation

To obtain the fluence rate distribution at the boundary of the sample, finite element solution of the diffusion equation has been used [3]. The diffusion equation could be expressed as [2]:

(14) $$\frac{\partial Ф\left(\vec{r},t\right)}{c\partial t}+\mu_{a}Ф\left(\vec{r},t\right)-\nabla .\left[D\nabla Ф\left(\vec{r},t\right)\right]=S\left(\vec{r},t\right)$$

where *D* is the diffusion coefficient which is defined as $D=\frac{1}{3\left(\mu_{a}+{\overset{‵}{\mu }}_{s}\right)}$, $Ф\left(\vec{r},t\right)$ is the fluence rate (in W/cm^2^) and $S\left(\vec{r},t\right)$ is the source term (in W/cm^3^ sr).

#### 2.3.3. Monte-Carlo Simulation

The Monte-Carlo method is a numerical approach to the transport equation that simulates the transport of the photon in turbid medium. This model is based on a random walk, where a photon or a photon package is traced through the tissue until it exits or until it gets entirely absorbed [21].

Generally, Monte-Carlo simulation assumes an infinitely narrow photon beam, perpendicularly incident on a multi-layered scattering medium. This pencil beam can be represented by an impulse (Dirac delta) function of space, direction, and time; thus, the responses are termed impulse responses or Green’s functions. Each layer is defined by its thickness, refractive index, absorption and scattering coefficients and scattering anisotropy [21].

The function that used in the simulation process are named MCML, the previously mentioned five parameters regarding the sample have to be known to use the simulation. The simulation output gives the value of diffuse reflectance that should be obtained from experimental measurements.

In our simulation problem, the obtained optical parameters and thickness of the samples have been introduced to MCML assuming matched boundary conditions to obtain the values of diffuse reflectance at each case, hence validating our results. Simulation were implemented under Matlab R2015a environment.

## 3. Results

**Figure 3:**
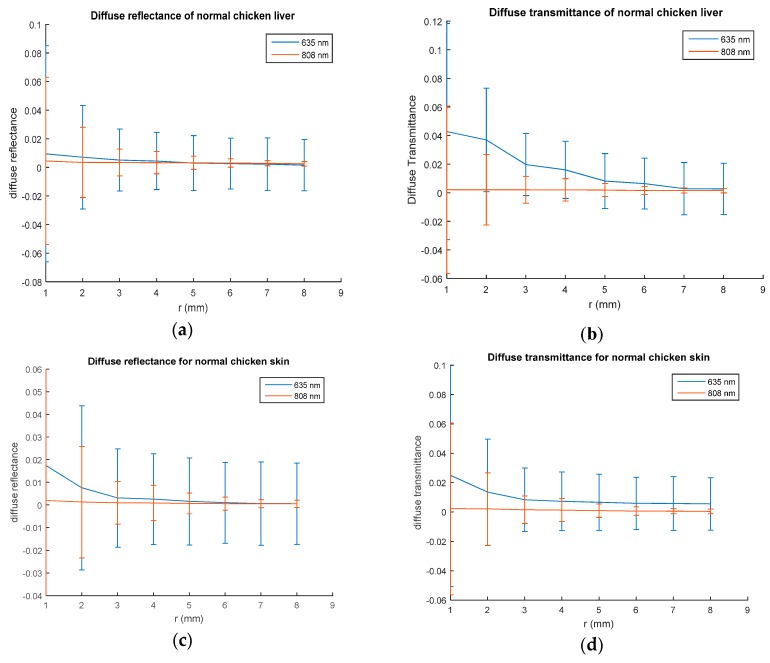
Spatially resolved steady state of: (**a**) diffuse reflectance of normal chicken liver; (**b**) diffuse transmittance of normal chicken liver; (**c**) diffuse reflectance of normal chicken skin; (**d**) diffuse transmittance of normal skin.

Using the proposed experimental setup, spatially resolved steady state diffuse reflectance and transmittance of normal chicken liver and normal breast chicken skin samples have been measured. The measurements were taken at different spatial distances from the laser source at the same line in the sample surface and repeated for the 635 nm and 808 nm laser. However, as it is an in vitro study, the experiments could be done for different tissue slices to get information about the inner parts of the sample. Figure 3 shows a plot of the measured diffuse reflectance and diffuse transmittance values for a distance r ranges from 1 to 8 mm. These measurements were taken almost five times at every distance, the standard deviation at each spatial step for each laser wavelength was calculated and presented in the figures through error bars.

**Figure 4:**
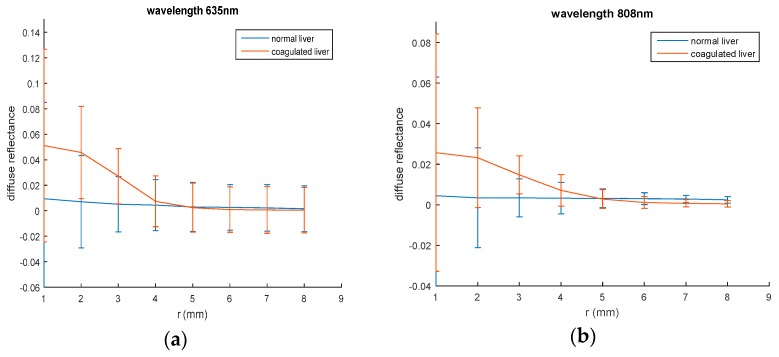
Spatially resolved steady state diffuse reflectance of normal and coagulated chicken liver: (**a**) at 635 nm; (**b**) at 808 nm laser irradiation.

To examine the heating effect of the samples on diffuse reflectance values, samples from same liver and chicken tissues have been boiled to get coagulated tissues. Spatially resolved diffuse reflectance measurements of a coagulated chicken liver were obtained at 635 nm and 808 nm laser irradiation using the same setup and the curve is presented in Figure 4.

The obtained measurements were then used to estimate the optical parameters of the samples at 635 nm and 808 nm lasers using a combination between Kubelka-Munk model and Beer-Lambert law and the results are presented in Table 1.

To validate our results, the measured diffuse reflectance values were verified using Monte-Carlo simulation [21] assuming refractive-index- matched boundary condition. The results are summarized in Table 2.

**Figure 5:**
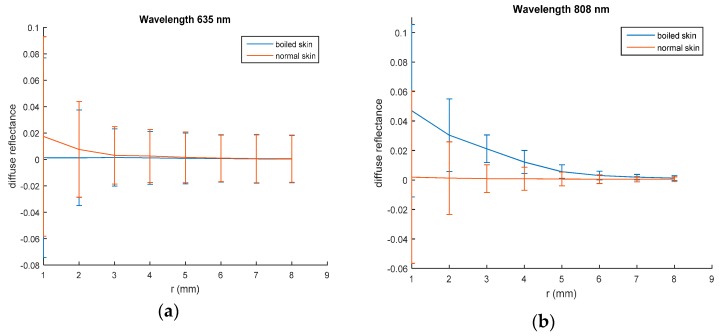
Spatially resolved steady state diffuse reflectance of normal and boiled breast chicken skin: (**a**) at 635 nm; (**b**) at 808 nm laser irradiation.

Diffuse reflectance curves in normal and boiled skin samples are illustrated in Figure 5.

**Figure 6:**
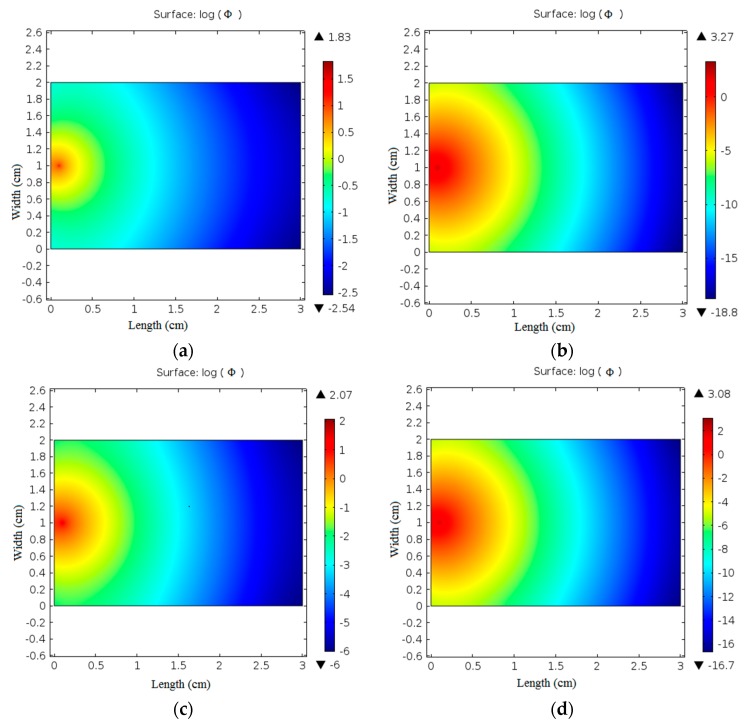
Fluence rate at tissue samples surface: (**a**) normal chicken liver at 635 nm; (**b**) coagulated chicken liver at 635 nm; (**c**) normal chicken liver at 808 nm; (**d**) coagulated chicken liver at 808 nm laser irradiation.

After optical parameters validation, the optical fluence rate distribution was simulated using finite element solution of the diffusion equation. The resultant fluence rate images of normal and coagulated chicken liver samples at 635 nm and 808 nm laser irradiation are presented in Figure 6.

**Figure 7:**
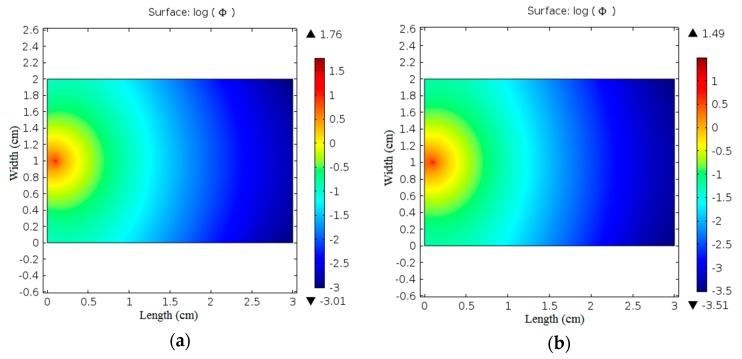

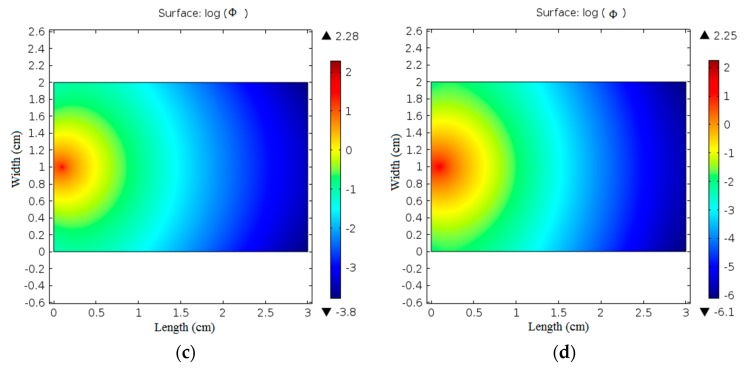
Fluence rate at tissue samples surface: (**a**) normal chicken skin at 635 nm; (**b**) boiled chicken skin at 635 nm; (**c**) normal chicken skin at 808 nm; (**d**) boiled chicken skin at 808 nm laser irradiation.

The obtained optical parameters values of normal and boiled skin were introduced to the diffusion equation of light propagation in biological tissue to investigate the change in the fluence rate distribution at tissue surface as a result of the change in the optical parameters. Figure 7 shows the difference in the optical fluence rate images in normal and boiled chicken skin samples when illuminated with 635 and 808 nm laser irradiation.

## 4. Discussion

The spatially resolved steady state diffuse reflectance and transmittance curves illustrated in Figure 3 show near exponential profiles which is normal for a light illuminating biological tissue. The maximum reflectance value appears at the first spatial step and decays when moving away from the light source. The curves also reveal the dependence of reflectance and transmittance values on the wavelength of the light source. Values of standard deviation presented in Table 1 show a maximum value at the first spatial step and also this value decays with increasing the moving step. This is due to avoiding the specular reflection and measurements that far enough from it becomes mainly diffuse.

These values were changed according to the tissue condition as shown in Figure 4; the maximum values of diffuse reflectance in the coagulated liver sample impressively increased compared with the value of normal sample at 635 nm and 808 nm. Changing sample conditions lead to many physiological changes regarding water and blood contents that typically affect absorption and scattering properties of the tissue.

The two wavelengths give almost the same behavior for normal and coagulated liver sample, while in normal and boiled skin, this behavior is changed as presented in Figure 5. However, when dealing with biological tissues, unexpected behaviors may occur. At 635 nm laser irradiation, the diffuse reflectance values of normal breast chicken skin are greater than those of boiled skin, while, boiled skin sample gives diffuse reflectance values more than normal skin at 808 nm. These results also reflect the dependence of tissues diffuse reflectance on the incident light wavelength.

Coagulated liver and boiled chicken skin samples were used to investigate the change in the optical parameters with the tissue condition. Moreover, two laser wavelengths in the near infrared range were used in that investigation, 635 nm and 808 nm to give appropriate results for light diffusion in biological tissues.

For performance evaluation, results from the literature were compared to the obtained results from the proposed measurements. Results reported by Hafeez-Ullah [22] on a 1 cm thick normal liver chicken at 810 nm pulsed laser showed a value of scattering coefficient equals to 2.6 mm^−1^ and absorption coefficient equals to 0.12 mm^−1^, while our obtained value of $\mu_{a}$ at this wavelength was 0.85 mm^−1^ and$\;\mu_{s}$ was 2.13 mm^−1^, the variance in $\mu_{a}$ could be due to different blood contents. No results were reported by [22] for 635 nm and no calculations were made to predict the anisotropy value. Instead, assumed value of g=0.8 was used.

Measurements obtained by [23] on breast chicken skin sample 8 mm thick at 635 nm using a double integrating sphere technique resulted in $\mu_{a}$ of 0.19 mm^−1^ and $\mu_{s}$ of 2.2 mm^−1^ our values were 0.25 mm^−1^ for $\mu_{a}$ and 1.6 mm^−1^ for $\mu_{s}$. The variance in values can be due to the difference in the sample thickness as the examined sample in this work was 1 mm thick that contains only the outer protection epidermis layer however, upon using 8 mm thick skin sample as presented in [19], the inner dermis layer and stratum corneum upper layer of epidermis will be included.

It should be mentioned here that the accuracy of predicting absorption and scattering parameters of a sample depends on the accuracy of measuring the total diffuse reflectance and transmittance. Therefore, Monte-Carlo simulation has been implemented to validate the obtained diffuse reflectance values as presented in Table 2. In chicken liver sample the absolute recorded error at 635 nm was 0.0083 and 0.0078 at 808 nm laser irradiation. While in coagulated liver, the values were 0.0148 and 0.0038 at 635 nm and 808 nm respectively. The absolute error in native skin was 0.009 at 635 nm and 0.0225 at 808 nm. In the boiled skin sample, the absolute error was 0.0191 and 0.0278 at 635 nm and 808 nm, respectively.

The calculated normal and coagulated chicken liver optical parameters values were used to obtain fluence rate distribution images at 635 nm and 808 nm laser as illustrated in Figure 6. The obtained images provide fine different distribution at each sample. In a normal liver, the maximum value of log(Ф) is 1.83 at 635 nm and 2.07 at 808 nm while this value is changed to 3.27 at 635 nm and 3.08 at 808 nm in the case of the coagulated liver. The minimum value of log(Ф) is also changed regarding the sample condition giving values of −2.54 and −6 for normal liver and −18.8 and −16.7 in coagulated liver at 635 nm and 808 nm respectively. The difference in fluence rate values at each wavelength resulting from the change in optical parameters values introduced to the diffusion equation to obtain the fluence rate distribution at sample’s surface. Varying the sample condition affects water and blood contents, and hence affects the values of its optical parameters.

Moreover, it can be observed from Figure 7 that the fluence rate distribution at the surface of the samples changes with the tissue type due to the change in their optical properties. With normal breast chicken skin tissue, 635 nm laser, the maximum value of log(Ф) is 1.76 and the minimum value is −3.01 while with boiled tissue the maximum value of log(Ф) is 1.49 and the minimum value is −3.51. Using 808 nm laser irradiation the maximum value of log(Ф) is 2.28 and the minimum value is −3.8 for normal skin tissue while the maximum value of log(Ф) changes to 2.25 and the minimum value is −6.1 for boiled skin tissue. These results reveal the significance of the obtained fluence rate distribution images in differentiation of biological tissue.

## 5. Conclusions

In conclusion, a method based on the optical fluence distributions on the tissue surfaces has been proposed as a new diagnostic tool. In this method, a combination of Kubelka-Munk model and Beer-Lambert law has been used to calculate the optical parameters of different samples of biological tissues from the measured values of diffuse reflectance and transmittance. Measurements were implemented using an experimental setup based on distant-detector. The proposed method for the moment is suitable for in vitro measurements, however, it could be upgraded via a usage of optical fiber probes to be suitable for in vivo study. The obtained results are promising since the fluence rate distribution images have discriminant features between different tissue types. Therefore, this method can be used in diagnosing and differentiating biological tissues.

## Figures and Tables

**Table 1:** The calculated optical parameters.

Sample	Wavelength	$\mu_{a}\left({\mathrm{cm}}^{-1}\right)$	$\mu_{s}\left({\mathrm{cm}}^{-1}\right)$	g
Normal chicken liver	635 nm	1.4129	24.7830	0.6794
	808 nm	8.5873	21.3700	0.7762
Coagulated chicken liver	635 nm	24.015	46.3455	0.3985
	808 nm	23.250	42.1177	0.5527
Normal chicken skin	635 nm	2.5433	16.4246	0.6117
	808 nm	2.3215	34.8758	0.6117
Boiled chicken skin	635 nm	4.9963	14.6141	0.8729
	808 nm	7.3333	54.3248	0.8371

**Table 2:** Monte-Carlo Validation.

Sample	Wavelength	Measured Values of Rd	Monte-Carlo Simulation Values of Rd
Normal Chicken liver	635 nm	0.0396	0.0469
	808 nm	0.0339	0.0422
Coagulated chicken liver	635 nm	0.1220	0.10713
	808 nm	0.0763	0.07244
Normal Chicken skin	635 nm	0.0343	0.0433
	808 nm	0.0072	0.0297
Boiled Chicken skin	635 nm	0.0071	0.0262
	808 nm	0.1225	0.0892
